# Specificity Analysis of Genome Based on Statistically Identical K-Words With Same Base Combination

**DOI:** 10.1109/OJEMB.2020.3009055

**Published:** 2020-07-14

**Authors:** Hyein Seo, Yong-Joon Song, Kiho Cho, Dong-Ho Cho

**Affiliations:** ^1^ School of Electrical EngineeringKorea Advanced Institute of Science and Technology (KAIST) 34968 Daejeon 300-010 South Korea; ^2^ Department of SurgeryUniversity of California 12218 Sacramento California 95064 USA

**Keywords:** Alignment-free, genetic algorithm, k-word, microbial pathogenicity, statistical specificity in k-word profile

## Abstract

*Goal:* Individual characteristics are determined through a genome consisting of a complex base combination. This base combination is reflected in the k-word profile, which represents the number of consecutive k bases. Therefore, it is important to analyze the genome-specific statistical specificity in the k-word profile to understand the characteristics of the genome. In this paper, we propose a new k-word-based method to analyze genome-specific properties. *Methods:* We define k-words consisting of the same number of bases as statistically identical k-words. The statistically identical k-words are estimated to appear at a similar frequency by statistical prediction. However, this may not be true in the genome because it is not a random list of bases. The ratio between frequencies of two statistically identical k-words can then be used to investigate the statistical specificity of the genome reflected in the k-word profile. In order to find important ratios representing genomic characteristics, a reference value is calculated that results in a minimum error when classifying data by ratio alone. Finally, we propose a genetic algorithm-based search algorithm to select a minimum set of ratios useful for classification. *Results:* The proposed method was applied to the full-length sequence of microorganisms for pathogenicity classification. The classification accuracy of the proposed algorithm was similar to that of conventional methods while using only a few features. *Conclusions:* We proposed a new method to investigate the genome-specific statistical specificity in the k-word profile which can be applied to find important properties of the genome and classify genome sequences.

## I. Introduction

The evolution of sequencing technology has generated a tremendous amount of genetic information [1]–[3]. Consequently, genome sequence analysis has been actively conducted to understand genome structure, phylogeny, disease, and other characteristics [4]–[7]. Sequence analysis techniques can be divided according to whether the alignment process [8], [9] is included or not included. Alignment-free methods analyze a sequence without the alignment procedure [10], [11]. These methods can be relatively inaccurate compared to alignment-based methods, but are less computationally complex and have advantages when considering long or multiple sequences.

The word count-based approach, which considers the consecutive *k* bases called a k-word or k-mer, is one of the most well-known alignment-free methods [12]–[20]. The feature frequency profile (FFP), which indicates the number of each possible k-word, was considered to analyze phylogeny [12], [13]. In [14]–[16], the location information of the k-word was extracted to improve the results of k-word-based methods. The spaced word, which defines “don't care” positions within a word pattern [17], has been utilized for different purposes in fast or accurate sequence comparisons [18]–[20].

In particular, certain studies have attempted to characterize the k-word profile of the genome sequence [21]–[24]. In [21], it was found that the k-word spectra had unimodal distribution in a majority of species. The nullomer, which is the k-word that does not appear in the sequence, was also studied in the human genome [22]. Based on Chargaff's rules [25]–[27], the symmetry properties of k-words were identified in [23], [24]. In [23], Chargaff's second rule [27] was generalized to demonstrate that reverse-complement words have a similar frequency. Moreover, the exceptional symmetry, meaning that the frequencies of reverse-complement words are similar, has been defined [24].

These previous studies are limited because they focused on the partial properties of the genome. The fundamental logic of the genome-specific k-word profile is that individual characteristics of the genome are determined by the base combination. The base combination is reflected in k-words, resulting in unique genome-specific statistical properties different from the random sequence in the k-word profile. In this paper, we statistically analyze the abnormalities of the k-word profile. We define statistically identical k-words (SIWs) as the k-words with the same number of bases. The ratio between frequencies of two SIWs (SIWR) is also defined and used to represent genome-specific statistical properties in the k-word profile. Furthermore, a search algorithm that modifies the genetic algorithm (GA) is suggested to determine a minimum set of SIWRs useful for genome classification. The proposed method is applied to the complete genome of microorganisms such as *Escherichia coli* (*E. coli*) strains and *Bacillus* species for microbial pathogenicity analysis.

## II. Materials and Methods

The combination of bases that make up the genome has an important role in determining the characteristics of the individual. Although the genome sequence may seem to be a random sequence of bases, it is actually the result of extremely complex principles. Fig. S1 in the supplementary material compared the difference between a random sequence and the actual genome sequence. The difference between the random sequence and actual genome sequence in the k-word profile occurs because complex base combinations determine the characteristics of the genome beyond the statistical prediction. Therefore, we suggest the analysis method of this genome-specific statistical specificity which is reflected in the k-word profile in order to characterize an individual genome.

### A. SIW

Because there are four types of bases $A$, $C$, $G$, and $T$, the number of k-words is ${4^k}$. Let the set of k-words be ${\boldsymbol{W}_k} = \{ {{w_{k,1}},{w_{k,2}}, \ldots,{w_{k,{4^k}}}} \}.$ Then, $i$-th k-word is ${w_{k,i}} = {w_{k,i}}(1){w_{k,i}}(2) \cdots {w_{k,i}}(k)$ for all $i \in \{ 1,2, \ldots,{4^k}$}, where ${w_{k,i}}(j) \in \{ {A,C,G,T} \}$ is the $j$-th element of ${w_{k,i}}$ for all $j \in \{ 1,2, \ldots,k$}. We define k-words with the same base combination as the SIWs, and the group of SIWs as the SIW set. When $a$, $c$, $g$, and $t$ represent the number of $A$, $C$, $G$, and $T$, respectively, the SIW set is written as $\boldsymbol{Z}_k^{a,c,g,t}$, where $0 \le a < k$, $0 \le c < k$, $0 \le g < k$, $0 \le t < k$, and $a + c + g + t = k$. If ${| {{w_{k,i}}} |_X}$ means the number of element $X$ among ${w_{k,i}}(1),{w_{k,i}}(2), \ldots,{w_{k,i}}(k)$, any k-word ${w_{k,i}}$ that satisfies the following conditions is an element of $\boldsymbol{Z}_k^{a,c,g,t}$:

\begin{equation*}
{\left| {{w_{k,i}}} \right|_A} = a,\ {\left| {{w_{k,i}}} \right|_C} = {\rm{c}},{\left| {{w_{k,i}}} \right|_G} = g,{\left| {{w_{k,i}}} \right|_T} = {\rm{t}}.\tag{1}
\end{equation*}

In the case of $k$, the number of SIW sets is equal to the number of $k$ selections from the four bases allowing for replication as follows:

\begin{equation*}
{\ _4}{\mathbb{H}_k} - 4 = _{\ 4 + k - 1}{\mathbb{C}_k} - 4 = \frac{{\left({4 + k - 1} \right)!}}{{k!3!}} - 4\tag{2}
\end{equation*}

where${\ _x}{\mathbb{H}_y}$ is the permutation with repetition selecting replicable $y\ $elements among *x* elements, ${_x}{\mathbb{C}_y}$ is the combination selecting $y$ elements among *x* elements, and $x! = x({x - 1})({x - 2}) \cdots 1$ is the factorial of *x*. K-words with only one kind of base such as $AA$, $CC$, $GG$, and $TT$ in the case of $k = 2$ are excluded from overall SIW sets because there are no other words configured with the same bases. On the other hand, the number of SIWs in the set $\boldsymbol{Z}_k^{a,c,g,t}$ is the number of cases listing all of $a$, $c$, $g$, and $t$ bases, which can be expressed with the multinomial coefficient as follows:

\begin{equation*}
\frac{{\left({a + c + g + t} \right)!}}{{a!c!g!t!}} = \frac{{k!}}{{a!c!g!t!}}.\tag{3}
\end{equation*}

### B. SIWR

As shown in Fig. S1, if the sequence is a random list of bases, k-word frequencies in the case of ${\rm{k}} > 1$ can be predicted from k-word frequencies in the case of ${\rm{k}} = 1$, and SIWs within an SIW set are expected to have a similar frequency. However, in the case of the genome, the prediction of k-word frequencies is difficult, and frequencies of SIWs may be different. The ratio between frequencies of any two SIWs within the SIW set is defined as SIWR. A large deviation of the SIWR value from “1” means that frequencies of the two SIWs are different from the statistical prediction. This indicates that these SIWRs represent the genome-specific k-word profile and are important in view of understanding the unique characteristics of the genome. Fig. S2 in the supplementary material illustrated an example of the SIWR calculation process.

Because the size of an SIW set follows (3), the number of SIWRs from the SIW set $\boldsymbol{Z}_k^{a,c,g,t}$ is calculated as

\begin{equation*}
{\ _{\frac{{k!}}{{a!c!g!t!}}}}{\mathbb{C}_2}.\tag{4}
\end{equation*}

Consequently, the total number of SIWRs in a specific $k$ is the summation of (4) for all $a$, $c$, $g$, and $t$ that satisfy $0 \le a < k$, $0 \le c < k$, $0 \le g < k$, $0 \le t < k$, and $a + c + g + t = k$ which can be written as:

\begin{equation*}
{M_k} = \mathop \sum \limits_{\begin{array}{c}\\
\scriptscriptstyle{\forall a,c,g,t}\\
\scriptscriptstyle{a + c + g + t = k}\\
\scriptscriptstyle{0 \le a < k,0 \le c < k,0 \le g < k,0 \le t < k} \end{array}}{_{\frac{{k!}}{{a!c!g!t!}}}}{\mathbb{C}_2}. \tag{5}
\end{equation*}

### C. Classification Threshold of SIWR

SIWR can investigate statistical abnormalities in the k-word profile of the genome and can be used for sequence comparison and classification. The data set consists of $N$ sequences and is divided into two groups: positive and negative. The SIWR matrix at $k$ is ${\boldsymbol{R}_k}$ and its element ${r_{k,mn}}$ refers to the $m$-th SIWR value of the $n$-th sequence for all $m \in \{ {1,2, \ldots,\ {M_k}} \}$ and $n \in \{ {1,2, \ldots,N} \}$*.* Then, the binary classification threshold $t{h_{k,m}}$ is defined as the reference value of the $m$-th SIWR at $k$ that supports the smallest error when classifying data into two groups using only the $m$-th SIWR. That is, each sequence can be classified as either a negative or positive group, depending on whether the $m$-th SIWR value of the sequence is greater than or less than $t{h_{k,m}}$. In this case, the real value $t{h_{k,m}}$ is determined by the value with the highest classification performance as follows:

\begin{equation*}
t{h_{k,m}} = \ \mathop {argmin}\limits_h T{P_{m,h}} + T{N_{m,h}}\tag{6}
\end{equation*}

where $T{P_{m,h}}$ and $T{N_{m,h}}$ indicate the number of correctly identified positive and negative elements, respectively, when $h$ is used as the classification threshold for the $m$-th SIWR. The $t{h_{k,m}}$ is not a standard value that can be applied to all data and is dependent on the property of the data set.

### D. Modified GA-Based Search Algorithm

Depending on the characteristics of the genome, the degree that the frequency of SIWs deviates from statistical predictions varies. Only some SIWRs are useful for data classification; thus, we need to find these important SIWRs. Furthermore, by combining important SIWRs, the classification performance can be improved over the case of single SIWR-based classification. Hence, we propose a GA-based search algorithm for identifying a minimum SIWR set which is optimized for classifying the genome data set [28].

**Fig. 1.**
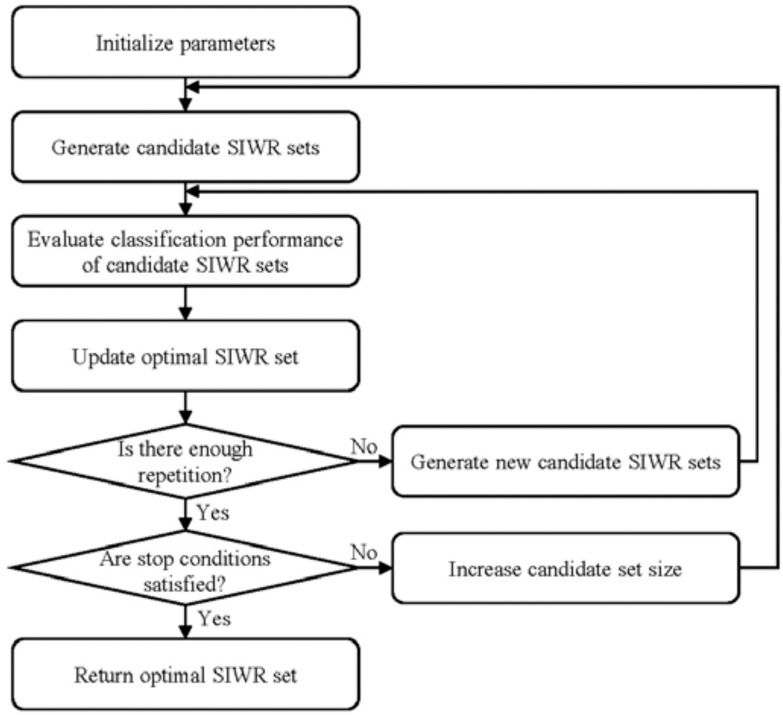
Flowchart of the proposed GA-based search algorithm.

Fig. 1 shows the flowchart of the proposed GA-based search algorithm to find the optimal SIWR set. First, we set the minimum and maximum numbers of SIWRs that make up the optimal set. The reason for limiting the optimal set size is to reduce the time required for the search because a small number of SIWRs can sufficiently categorize the entire data set. Then, we generate candidate sets consisting of the minimum number of SIWRs and evaluate the classification performance of each candidate set. The candidate set classifies the data following the majority voting system. That is, each SIWR of the candidate set classifies the data according to its classification threshold, and the candidate set classifies the data according to the classification result of more than half of the member SIWRs. If there is a superior candidate set, the optimal set is updated. The optimal set update is sufficiently repeated with the new candidate sets generated through selection, crossover, and mutation similar to the natural selection process. Then, when it is determined that the optimal set can completely classify the genome data, the algorithm is terminated. Otherwise, we increase the size of the optimal set and repeat the algorithm up to the maximum number of SIWRs. Algorithm S1 in the supplemental material displays the pseudo-code of the proposed GA-based search algorithm.

## III. Results

### A. Sequence Data Sets

Table SI and Table SII in the supplementary material present the name, accession number, and pathogenicity of the *E. coli* strains and *Bacillus* species sets. All of the complete genome sequences can be obtained from GenBank [29]. We assigned “pathogenic” as the positive group and “non-pathogenic” as the negative group. In addition, we perform the pathogenicity classifications and calculate accuracy ($Ac$), prediction ($Pr$), and recall ($Re$).

### B. Pathogenicity Classification Using Single SIWR

**Fig. 2.**
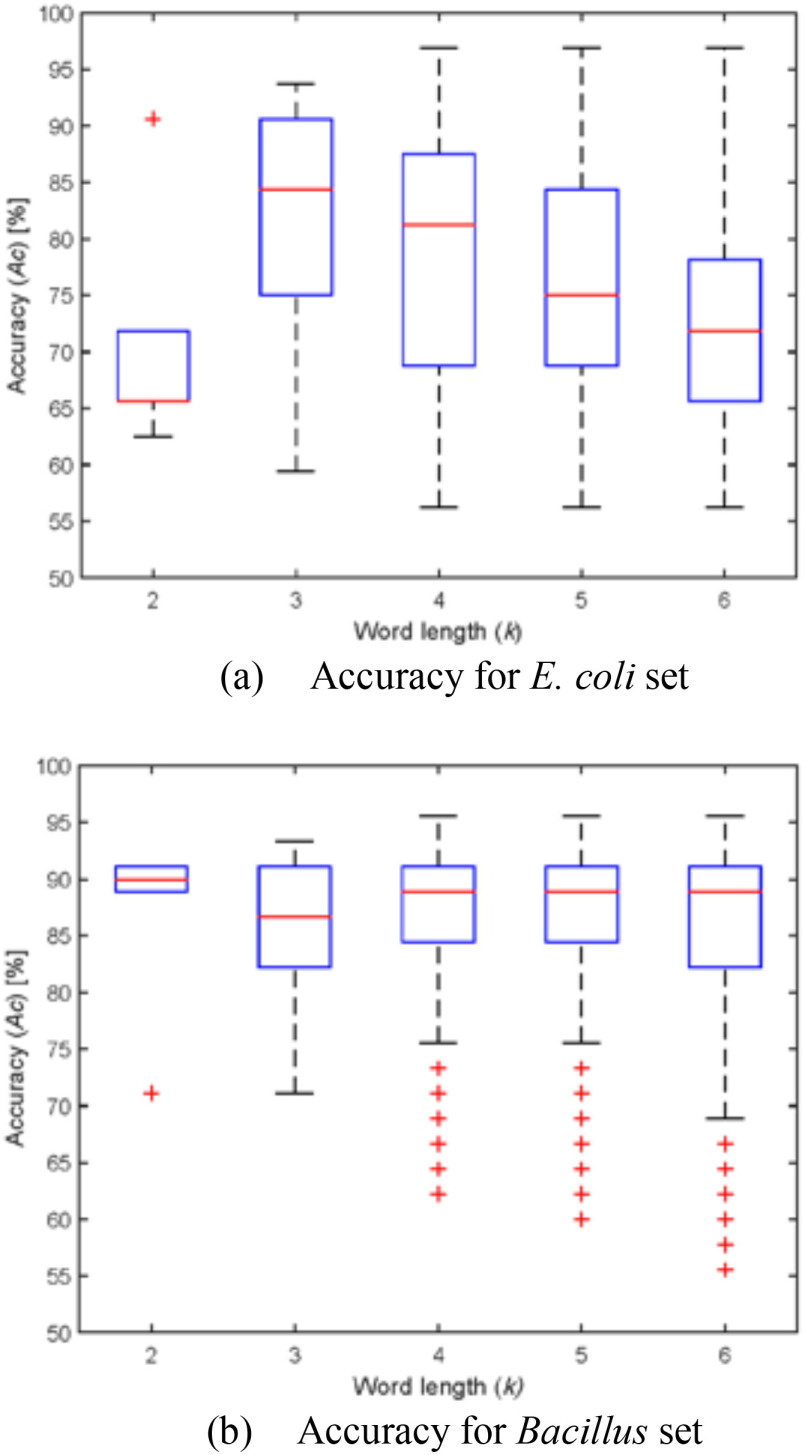
Accuracy of single SIWR-based classification for the *E. coli* and *Bacillus* sets.

Following (6), $t{h_{k,m}}$ was determined to be the best value distinguishing the *E. coli* and *Bacillus* sets. Fig. 2(a) and Fig. 2(b) summarize the distribution of the classification performance of the SIWRs using their $t{h_{k,m}}$ for the *E. coli* strains and *Bacillus* species data sets, respectively. The $Ac$ value of all SIWRs was represented as a box plot in the case of $2 \le {\rm{k}} \le 6$. The median value was drawn with the red-central line, and the blue-box contained values from the 25th percentile to 75th percentile. In the *Bacillus* set, the $Ac$ value was typically larger than in the *E. coli* set, and the majority of the SIWRs demonstrated similar performance to the median value. The pathogenicity determination is the inter-species classification in the *E. coli* set and inter-genus classification in the *Bacillus* set. Because of this difference, the pathogenicity classification results for the *Bacillus* species were relatively more accurate. However, SIWRs with low performance also appeared in the *Bacillus* set. Although the majority of the SIWRs in the *E. coli* set indicated degraded performance compared to the *Bacillus* set, the best-performing SIWR indicated an above 90% $Ac$ value for both data sets.

### C. Pathogenicity Classification Using SIWR Set

To evaluate the performance of the proposed GA-based SIWR set search algorithm, which is represented as *SIWR search*, we considered three conventional methods: *FFP tree*, *FFP svm*, and *Spaced svm*. For the case of *FFP tree* [12], [13], we used a phylogeny tree by applying a neighbor-joining method [30] and Euclidean distance for FFP. In the case of *FFP svm* and *Spaced svm* [20], we applied SVM [31] to obtain the best performance of conventional FFP and spaced word with one “don't care” position, respectively. All sequences were used as training data and the classification performance after training completion was evaluated.

**Fig. 3.**
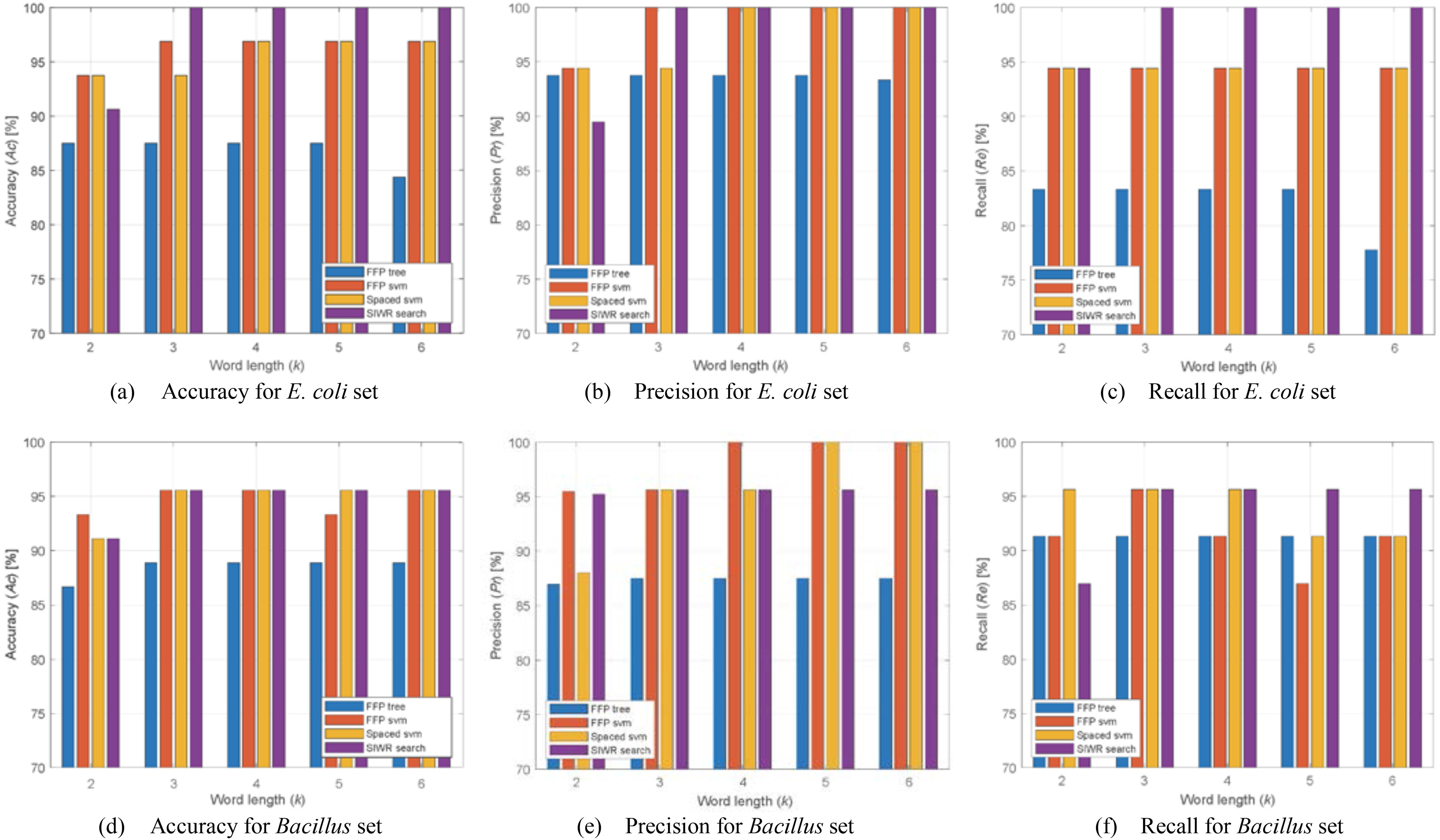
Comparison of classification performance of the proposed and conventional methods for *E. coli* and *Bacillus* sets.

The pathogenicity classification performances for *E. coli* and *Bacillus* sets were compared in Fig. 3, and the number of used features were compared in Table I. We confirmed that the classification performance of the FFP method increased when using SVM (*FFP svm*) rather than tree generation (*FFP tree*). Compared to *FFP svm* and *Spaced svm*, the proposed *SIWR search* achieved similar or superior classification performances for the *E. coli* set. In particular, *SIWR search* identified all the sequences in the case of $3 \le k \le 6$. For the *Bacillus* set, *SIWR search* was able to achieve acceptable performances similar to *FFP svm* and *Spaced svm*. Therefore, *SIWR search* had the advantage of performing efficient classification using very few features compared to other methods. Unlike the *E. coli* set, the *Bacillus* set was composed of different species, making it difficult to determine common information from the variety of pathotypes. The proposed GA-based search algorithm could obtain an SIWR set with an extremely small number of member SIWRs, which is useful for pathogenicity classification. Since the proposed method assigns the sequence group by a group determined by a majority of member SIWRs, the group determination is difficult when an even number of member SIWRs is selected. Therefore, the number of selected SWIRs was odd.

**TABLE I:** Comparison of Number of Features of Proposed and Conventional Methods for *E. Coli* and *Bacillus* Sets

E. coli	$k = 2$	$k = 3$	$k = 4$	$k = 5$	$k = 6$
FFP	16	64	256	1,024	4,096
Spaced	8	48	256	1,280	6,144
Proposed	1	5	3	3	3
*Bacillus*	$k = 2$	$k = 3$	$k = 4$	$k = 5$	$k = 6$
FFP	16	64	256	1,024	4,096
Spaced	8	48	256	1,280	6,144
Proposed	1	3	1	1	1

## IV. Discussion

### A. Base Composition of Significant SIWRs

**Fig. 4.**
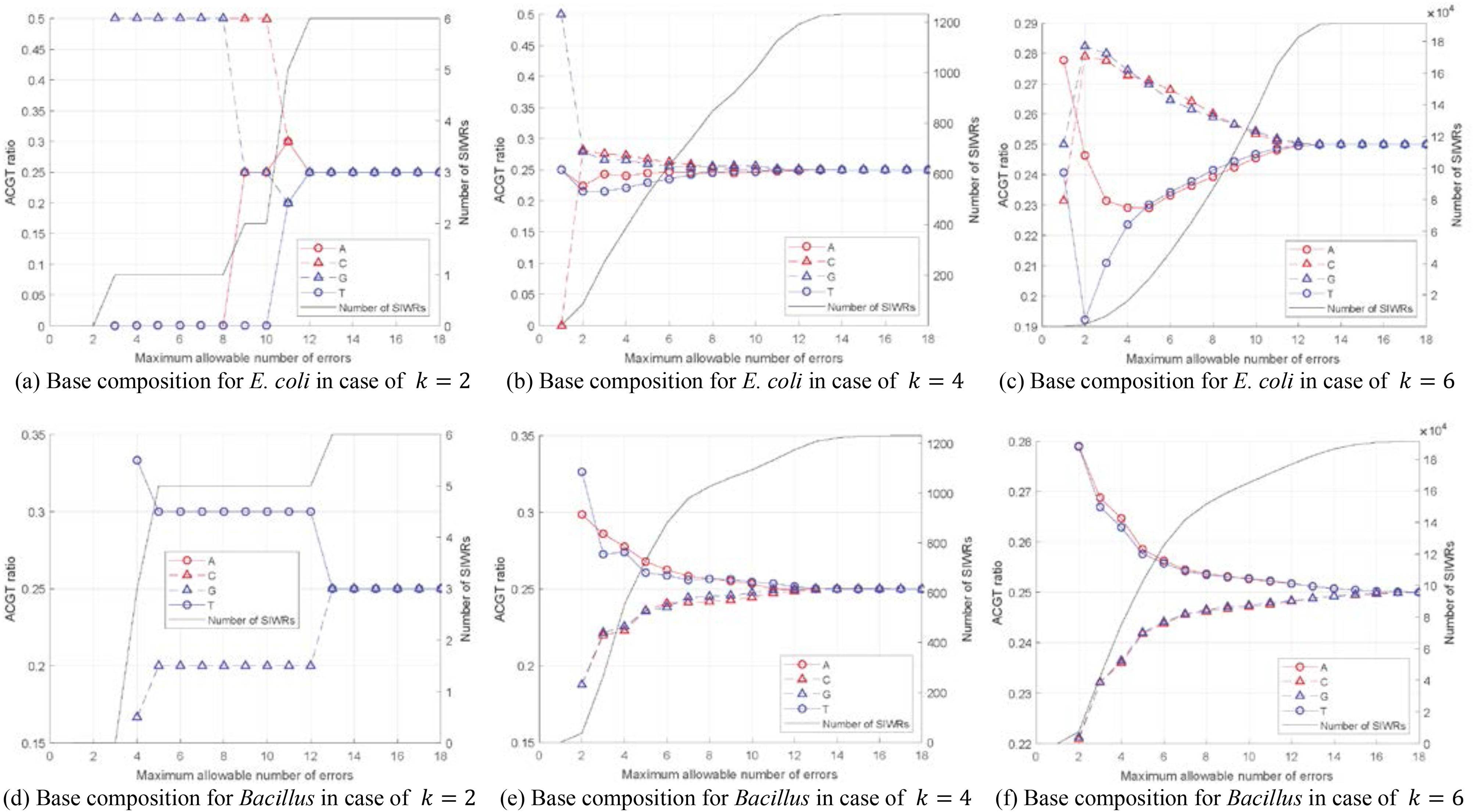
The number of significant SIWRs and base combination of significant SIWRs for the *E. coli* and *Bacillus* sets.

In this section, the bases that were included in the useful SIWRs for the microbial pathogenicity classification are discussed. We define the significant SIWRs as the SIWRs that satisfy the maximum allowable number of errors when classifying data with their thresholds $t{h_{k,m}}$. Fig. 4 showed the proportion of bases existing in the significant SIWRs in the case of $k \in \{ {2,\ 4,\ 6} \}$ for the *E. coli* and *Bacillus* sets. The number of significant SIWRs was also illustrated.

As shown in Fig. 2, the average classification accuracy was higher in the *Bacillus* set than in the *E. coli* set, and there were more significant SIWRs in the *Bacillus* set than in the *E. coli* set when they have the same $k$ in Fig. 4. For the *E. coli* set, $C$ and $G$ appeared more frequently in significant SIWRs than $A$ and $T.$ In contrast, there were more $A$ and $T$ in significant SIWRs for the *Bacillus* set. This tendency gradually disappeared as the number of maximum allowable errors is increased and the number of non-significant SIWRs increased in the selected SIWRs. Curiously, the ratio of $A$ and $T$ were relatively high when only a small number of errors are allowed in the *E. coli* set. It is interesting that the significant SIWRs useful in the pathogenicity classification of the *E. coli* and *Bacillus* sets consisted of different base compositions. Moreover, the crossing of the base ratios in the *E. coli* set seems to be an unusual phenomenon. Therefore, we hope that further research on significant SIWRs can provide a deeper understanding of microbial pathogenicity and other phenotypes.

### B. Common Microbial Pathogenicity

The common microbial pathogenicity was analyzed by integrating the *Bacillus* and *E. coli* sets and classifying them on the basis of pathogenicity using the proposed method. Fig. S3 to Fig. S5 and Table SIII in the supplementary material showed the pathogenicity analysis results of the merged *E. coli* and *Bacillus* data sets. Although most SIWRs resulted in relatively lower $Ac$ values compared to the classification of single data sets, there were also some SIWRs resulting in a high $Ac$ of more than 90% for the merged data set (Fig. S3). It was also found that the number of SIWRs generally useful for pathogenicity classification in the merged *E. coli* and *Bacillus* data sets is fewer than the number of SIWRs useful for each data set classification. However, using the proposed GA-based search algorithm, we could find a set of SIWRs that resulted in good classification performance even in the merged data set (Fig. S4). Compared with conventional methods, *SIWR search* was able to attain a similar or better pathogenicity classification performance using fewer features (Table SIII). Because the increase of the number of significant SIWRs was slow, we could infer that the classification of pathogenicity in the merged data set was more difficult than in single data sets (Fig. S5). Generally, there were numerous $A$ and a relatively smaller number of $C$ and $G$ in significant SIWRs. In the case of the single data set, $C$ and $G$ or $A$ and $T$ appeared equitably in significant SIWRs, whereas there are more $A$ than $T$ in the merged set.

## V. Conclusion

In this study, we proposed a new k-word-based alignment-free sequence analysis method to evaluate the genome-specific properties reflected in the k-word profile. To analyze the difference between statistical expectation and actual k-word frequency, SIWR was defined and used for sequence classification. Moreover, a GA-based search algorithm was introduced to determine the minimum SIWR set required to classify a specific data set. We applied SIWR analysis for microbial pathogenicity identification for E. coli strains and Bacillus species.

In a future study, we want to classify sub-groups of E. coli strains and Bacillus species with regard to certain phenotypes that are more specific than the pathogenicity classification. Our study can also be extended to other pathogens to compare the degree and type of pathogenicity and investigate their general characteristics. In particular, the analysis of pandemic viruses such as SARS-CoV, MERS-CoV, and COVID-19 virus (SARS-CoV-2) is an interesting application of the proposed method. Additionally, because the selection of the optimal k-word size is an important issue in k-word-based methods, we would like to develop a method to select the optimal size of k-word suitable for SIWR analysis. Moreover, SIWR can be utilized along with various k-word methods such as spaced word, word position, and word interval. However, an additional computational complexity of $\mathcal{O}({\boldsymbol{N}{\boldsymbol{M}_{\boldsymbol{k}}}})$ for calculating the SIWR values is required. Furthermore, we can evolve the proposed method to perform multi-class classification using multiple classifiers.
